# Intracranial haemorrhage detected by cerebral computed tomography after falls in hospital acute medical wards

**DOI:** 10.1186/s12913-019-4634-8

**Published:** 2019-11-04

**Authors:** Shiny Stephen, Elena W. W. Wong, Adam M. Idris, Andy K. H. Lim

**Affiliations:** 1 Department of General Medicine, Monash Health, Clayton, Victoria 3168, Australia; 2 Department of Medicine, Monash University, Clayton, Victoria 3168, Australia; 3 Dandenong Hospital, Monash Health, 135 David Street, Dandenong, Victoria 3175 Australia

**Keywords:** Falls, Hospitals, Inpatients, Intracranial haemorrhage, Computed tomography, Neuroimaging

## Abstract

**Background:**

There is little published data on brain imaging and intracranial haemorrhage after hospital inpatient falls. Imaging protocols for inpatient falls have been adopted from head injury guidelines developed from data in patients presenting to the Emergency Department. We sought to describe the use of brain computed tomography (CT) following inpatient falls, and determine the incidence and potential risk factors for intracranial haemorrhage.

**Methods:**

We identified inpatient falls in acute medical wards at Monash Health, a large hospital network in the southeast region of Melbourne in Australia, from the incident reporting system during a 32 month period. We examined the post-fall medical assessment form, neurological observation chart and the diagnostic imaging system for details of the fall and brain CT findings. We used survival analysis to evaluate the timeliness of brain imaging and determined potential risk factors for intracranial haemorrhage by logistic regression.

**Results:**

From 934 falls in 789 medical inpatients, 191 brain CT scans were performed. The median age of patients was 77 years. Only 55% of falls were from standing height and 24% experienced a head strike. Less than 10% of patients received an urgent scan within one hour, and timeliness of imaging was influenced by anticoagulation status rather than guideline determination of urgency. The overall incidence of intracranial haemorrhage was 0.9%. The factors associated with intracranial haemorrhage were head strike, anticoagulation, loss of consciousness or amnesia, drop in Glasgow Coma Scale and advanced chronic kidney disease.

**Conclusions:**

The incidence of intracranial haemorrhage was low as most inpatient falls were at low risk for head injury. Research is needed to determine if guidelines specific for hospital inpatients may reduce unnecessary scans without compromising case detection, and improve timeliness of urgent scans.

## Background

The World Health Organisation (WHO) defines a fall as “*an event which results in a person coming to rest inadvertently on the ground or floor or other lower level*.” [1] In a 2015 national audit in the United Kingdom, the incidence of inpatient falls was estimated at 6.6 per 1000 bed days, with 0.19 per 1000 resulting in moderate or severe harm, or death [2]. In the United States, the incidence of inpatient falls was estimated as 3.6 per 1000 bed days, with 26% resulting in injuries or death [3]. In Australia for 2015–2016, inpatient falls occurred in over 34,000 separations, giving an estimated rate of 4.6 per 1000 separations for public hospitals [4]. Inpatient falls result in prolonged hospitalisation and additional health care costs [5]. However, one of the most dreaded complications is intracranial haemorrhage (ICH).

The risk of ICH may be different for hospitalised patients compared to head injury acquired in the community. Firstly, the mechanism of injury in hospitalised patients is mostly low impact or ground-level falls, rather than high impact injuries which are more common in the community [6]. Secondly, there is usually minimal delay between injury and medical assessment in hospital wards as most patients are under regular nursing observation. Thirdly, a hospital fall does not usually determine disposition of the patient for the next 24–48 h, unlike in the Emergency Department (ED), where imaging may facilitate a timely discharge [7].

Computed tomography of the brain (CTB) is the first-line imaging for assessing intracranial injury within the first 24 h [8]. Various clinical decision rules exist to guide the use of CTB in suspected intracranial injury in patients presenting to ED. They include the Canadian CT head rules (CCTHR) [6], New Orleans criteria [7], National Emergency X-Radiography Utilisation Study II criteria (NEXUS II) [8], and the National Institute for Health and Care Excellence (NICE) guidelines [9], which are based on the CCTHR. The CCTHR, which is the most widely validated, was developed using data from 3121 patients across 10 Canadian communities, based on risk factors considered to be high or medium risk for predicting the need for neurosurgical intervention. Factors in the criteria considered high risk were 100% sensitive and 68.7% specific, while the medium-risk factors had a sensitivity of 98.4% and specificity of 49.6% [6]. The NICE guidelines are similar to the CCTHR, but additionally recommend performing imaging for patients with a history of bleeding or clotting disorders [9].

The incidence of ICH in patients presenting to the ED with community falls is variable. Studies using the CCTHR to guide the use of CTB in community-fallers presenting to the ED with mild head injury showed that less than 10% have an ICH and less than 1% required neurosurgical intervention [6–8, 10, 11]. There is limited data on the incidence of ICH after a fall in hospital, with only one available study suggesting a much lower rate of 1.2% over four years, among 933 inpatients with a median age of 65 years [12]. There are no studies to date assessing the indications for CTB in an inpatient setting. Currently available decision-making tools for head injury assessment used in the ED have not been validated for inpatient falls. It is unclear if existing community head injury guidelines can be generalised to inpatient falls.

Due to potential differences between community and inpatient falls, it is also unclear if previously examined risk factors for ICH in community studies carry similar weight for inpatient falls. Such established risk factors for ICH in community studies include a history of hypertension, antiplatelet and anticoagulant medication use, and neurological symptoms (vomiting, amnesia, headache and loss of consciousness) [13–15]. Some factors such as age and dementia remain controversial [10, 14, 16]. Other potential risk factors remain theoretical but have a physiological basis for concern. This includes platelet dysfunction and abnormal platelet-vessel wall interactions which enhance bleeding risk in advanced chronic kidney disease (CKD) [17, 18]. Use of non-steroidal anti-inflammatory drugs also affect platelet function and haemostasis [19]. Chronic liver disease can result in a reduction in procoagulant factors, thrombocytopaenia and hyperfibrinolysis [20]. Any cause of coagulopathy or thrombocytopaenia may enhance bleeding risk. It has also been suggested that obesity may influence the head injury incidence and severity following blunt trauma [21].

This study aimed to: (1) characterise inpatient falls leading to assessment of head injury in acute medical wards, (2) determine the incidence of ICH post fall, (3) evaluate factors associated with ICH post fall, and (4) describe the rate of utilisation of CTB post fall and compliance with adopted guidelines.

## Methods

### Study design and setting

We conducted a retrospective study of falls in acute medical inpatients at two major referral centres at Monash Health in Victoria, Australia (Monash Medical Centre, Dandenong Hospital), from 15 Feb 2015 to 30 Sep 2017. Monash Health is a large tertiary hospital network in the south-east region of Melbourne, Australia. It is the largest public health service in the state of Victoria, providing healthcare to one quarter of Melbourne’s population. The majority of the high acuity medical care is provided at Monash Medical Centre and Dandenong Hospital, so this study did not include patients in the rehabilitation, aged care, mental health, regional and community health centres of the network which do not have an intensive care unit. Annually, the health service handles more than 260,000 hospital admissions.

### Patient selection and source

We identified eligible adult patients who had a fall from the RISKMAN database, a centralised electronic reporting system introduced by the Victorian State Department of Health for incident reporting in public health services. All health professionals are trained in use of this system on commencement of work at a health organisation, and mandated to report incidents, including falls. While details of the incident are often entered into the database, for the purpose of our study, we only used the RISKMAN system to identify patient falls and the timing of the incident. The remaining details of the fall were obtained from the information in the medical records and post-fall assessment form. The inclusion and exclusion criteria are shown in Table 1.

**Table 1:** Study inclusion and exclusion criteria

Inclusion criteria	Exclusion criteria
Patients ≥18 years old.	Patients < 18 years old.
Patients admitted to an acute medical inpatient ward.	Patients admitted to the day procedure unit, Emergency department short stay, non-medical ward or hospital-in-the-home. Falls outside the patient’s ward, such as cafeteria.
Falls reported to the centralised electronic incident reporting system (RISKMAN Database).	Poor documentation: Falls with inadequate or missing documentation in the patient medical record or post-fall medical assessment form.
Events meeting the World Health Organisation (WHO) definition for a fall.	Events not meeting the WHO definition for a fall: 1. Near-misses, where a patient was aided or assisted to a lower level, or prevented from reaching the ground. 2. Patient witnessed to intentionally lower themselves to ground, such as from fatigue or dyspnoea.

### Outcomes and study variables

The main outcome of interest was ICH. Monash Health introduced a formal post-fall assessment form in 2012 (Additional file 1: Figure S1). This form was adapted from the NICE guidelines and was designed for assessment of patients after falls in hospital to guide decision-making for brain imaging. From the medical record, post-fall medical assessment form, neurological observation chart and diagnostic imaging system, we extracted data on demographics, body mass index (BMI), comorbidities, antithrombotic and anticoagulant use, haematology and biochemistry profile, falls event and CTB findings.

We defined advanced CKD as a baseline estimated glomerular filtration rate (eGFR) less than 30 ml/min/1.73m^2^. Acute kidney injury was defined as a serum creatinine increase of over 50% from baseline within the last 7 days, or an increase by 27 μmol/L over the last 48 h, per the Kidney Disease: Improving Global Outcomes (KDIGO) criteria [22]. A platelet count of less than 50,000/mL was considered significant thrombocytopaenia, and an International Normalised Ratio (INR) ≥1.4 in the absence of anticoagulation as evidence of coagulopathy. The following dichotomous variables were determined from the clinical history: dementia, delirium, diabetes, hypertension and liver cirrhosis. Patients with active cancer were divided into mutually exclusive categories based on intracranial involvement. A remote history of cancer was disregarded. BMI was calculated as weight (in kilograms) divided by the height (in metres squared). Obesity was defined as a BMI of 30 kg/m^2^ of greater.

We determined indications for CTB based on the NICE guidelines (not indicated, urgent and non-urgent), as adopted by Monash Health (Additional file 1: Figure S1). We determined the imaging interval as the interval between the fall event (time recorded by first responder) and the time CTB was performed. To determine the outcomes following CTB, we interpreted the clinical management plan of the treating team after noting the CTB result (no change in treatment, medication change, surgical intervention, palliative therapy or transfer).

### Statistical analysis

Analysis was performed with STATA version 15 (StataCorp, TX, USA). A *P* < 0.05 was considered statistically significant. We used the Wilcoxon rank-sum test to compare the distribution of continuous data between groups. We used Fisher’s exact test to examine the association between CTB findings and clinical outcomes, and between CKD status and anticoagulation. We applied survival analysis for the imaging interval, and regard the endpoint (outcome) as the time CTB was performed. For the survival analysis, we report the log-rank statistics for the univariable analysis and used the Cox proportional hazards model to examine the effect of multiple independent variables on the outcome.

We determined the association between potential risk factors and ICH by logistic regression. To determine the potential effect of multiple falls for some patients, a mixed-effects logistic model was used to allow for the clustering of falls. We then used a likelihood ratio test to examine the residual between cluster variance, to determine if a multilevel model was required. If the residual between cluster variance was not significantly different from zero, we fit a standard logistic regression model and used the cluster-robust variance estimator to determine the standard errors.

## Results

### Patient characteristics

**Fig. 1:**
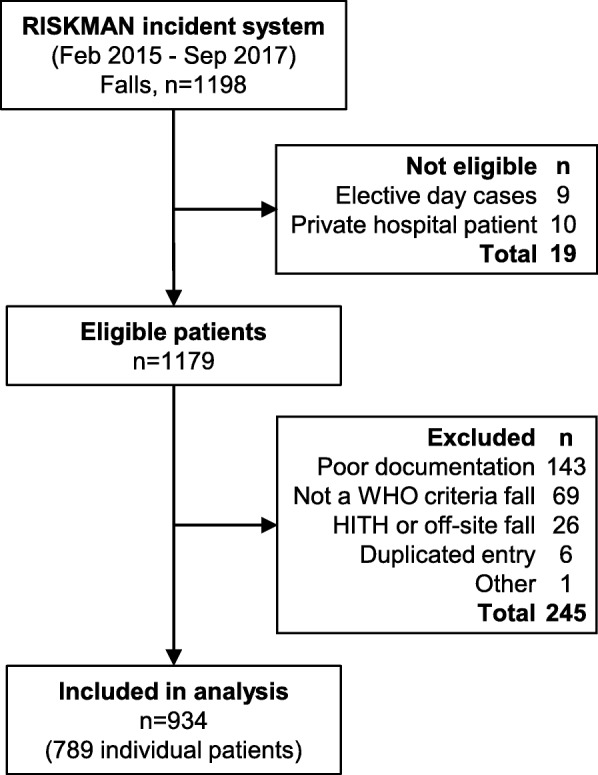
Study flow diagram, showing patient selection and exclusions. Footnote: HITH = Hospital in the home

From the 1198 falls identified, we included 934 falls occurring in 789 individual patients in the final analysis. The reasons for exclusion are shown in the study flow diagram (Fig. 1). The demographic, clinical characteristics and antithrombotic medication use of the included patients are summarised in Table 2. The median age was 77 years, with 18% affected by pre-existing dementia, and 25% experienced an acute delirium. Half the patients had chronic hypertension, one third was diabetic, and 59% were on antiplatelets or anticoagulants. Body mass index could not be calculated in 174/789 (22.1%) of patients. In the remainder, half were overweight and one quarter were obese.

**Table 2:** Demographics, comorbidities and antithrombotic medication use (*N* = 789)

Characteristic	*n* (%)
*Demographics*	
Median age (interquartile range) in years	77 (66–84)
Male sex	445 (56.4)
*Comorbidities*	
Diabetes	276 (35.0)
Hypertension	414 (52.5)
Cancer	
No intracranial involvement	123 (15.6)
Intracranial involvement	19 (2.4)
eGFR< 30 ml/min/1.73m^2^	83 (10.5)
Liver cirrhosis	43 (5.8)
Coagulopathy INR ≥ 1.5	31 (3.9)
Platelet count < 50,000/μL	15 (1.9)
Cognitive impairment	
Dementia	144 (18.3)
Intellectual disability or ABI	27 (3.4)
Delirium	201 (25.4)
Obesity^a^	158 (25.7)
Median BMI (interquartile range) in kg/m^2^	25.6 (21.5–30.4)
*Medications affecting coagulation*	
Single antiplatelet	245 (31.1)
Dual antiplatelet	51 (6.5)
Anticoagulants	
Warfarin	65 (8.2)
Novel oral anticoagulants^b^	67 (8.5)
Heparin (therapeutic dosage)	35 (4.4)
Non-steroidal anti-inflammatories	22 (2.8)

*ABI* Acquired brain injury, *BMI* Body mass index

^a^Data missing in 22.1% of patients

^b^apixiban, rivaroxaban, or dabigatran

### Antithrombotic medications

The proportion of patients receiving anticoagulants was not statistically different between those with advanced CKD and those without (26.0 vs. 19.2%, *P* = 0.14). However, the choice of anticoagulant was different between patients with and without advanced CKD (*P* < 0.001), as shown in Table 3. Compared to patients without advanced CKD, the proportion of patients receiving warfarin was much higher in those with advanced CKD. Concomitantly, the use of novel oral anticoagulants was lower with advanced CKD. The use of therapeutic heparin was proportionally similar. There were more patients with advanced CKD receiving antiplatelet treatment compared to those without advanced CKD (47.9 vs. 35.5%, *P* = 0.019).

**Table 3:** Antiplatelet and anticoagulation medication use by chronic kidney disease status (*N* = 934)

Medication	eGFR ≥30 *n* (%)	eGFR < 30 *n* (%)
Anticoagulant		
None	677 (80.8)	71 (74.0)
Warfarin	53 (6.3)	18 (18.7)
NOAC	77 (9.2)	3 (3.1)
Therapeutic enoxaparin	31 (3.7)	4 (4.2)
Antiplatelet		
None	541 (64.6)	50 (52.1)
Single	250 (29.8)	35 (36.5)
Dual agent	47 (5.6)	11 (11.5)

*NOAC* apixaban, rivaroxaban, dabigatran

*eGFR* estimated glomerular filtration rate in ml/min/1.73m^2^

### Falls characteristics

**Fig. 2:**
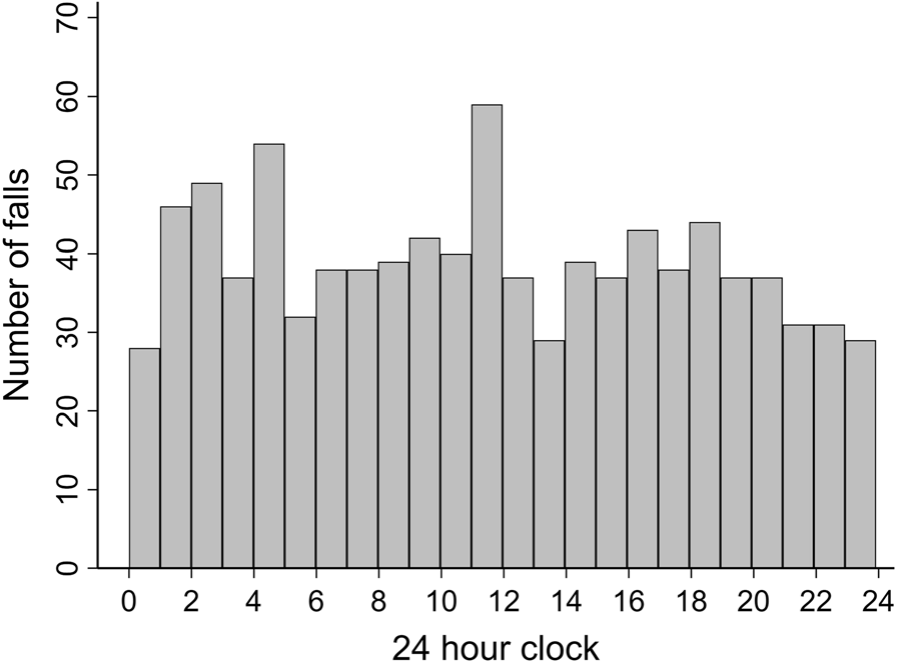
Frequency distribution of inpatient falls by the hour of day (*n* = 934)

The timing of falls was uniformly spread across a 24-h period, with no apparent differences between nursing shifts (night vs. day) or significant peaks to suggest any critical time period of high risk for falls (Fig. 2). The characteristics of the falls and the neurological outcomes are summarised in Table 4. Approximately 45% of the falls were considered low risk, such as falls out of bed or a sitting position, and 70% occurred without a suspected head strike. However, 70% of falls were unwitnessed and a formal Glasgow Coma Scale (GCS) score was not available for 56/934 (6.0%) of falls.

**Table 4:** Falls characteristics and post-fall neurological assessment (*N* = 934)

Characteristic	*n* (%)
Fall height	
Standing/ambulating	510 (54.6)
Sitting	167 (18.0)
Fall out of bed	245 (26.2)
Other	12 (1.2)
Witnessed fall	274 (29.3)
Head strike	222 (23.8)
Lost consciousness or amnesia	26 (2.8)
Presence of distracting injury	63 (6.8)
Drop in GCS > 2 points^a^	29 (3.1)
Neurological symptoms or deficit	48 (5.1)
New or worsening confusion	22 (2.4)
Focal deficit	10 (1.1)
Severe headache	5 (0.5)
Vomiting	1 (0.1)
Seizure	6 (0.6)
More than 2 symptoms/signs	4 (0.4)

*GCS* Glasgow Coma Scale

^a^Data missing in 6.0% of falls

### Cerebral imaging

A total of 191 CTB were performed, representing 20.5% of falls. The average imaging interval by clinical indication and urgency are shown in Table 5. Only 6.6% of urgent CTB were done within one hour and 62.3% within four hours. However, around 80% of CTB were performed within eight hours. In the survival analysis, there was no statistical difference in the imaging intervals between the urgent, non-urgent and non-indicated CTB (χ^2^ = 3.67, df = 2, *P* = 0.16). There was weak evidence that imaging intervals were longer for non-indicated CTB compared to indicated CTB (χ^2^ = 3.57, df = 1, *P* = 0.059).

**Table 5:** Brain CT imaging intervals by indication and clinical urgency (*N* = 191)

Indication and urgency	Total number of patients *n* (%)	Imaging interval in hours Median (IQR)	CTB within one hour *n* (%)	CTB within eight hours *n* (%)
Overall	191 (100.0)	3.7 (2.2–6.6)	6 (3.1)	154 (80.6)
Urgent	61 (31.9)	3.2 (1.9–5.3)	4 (6.6)	49 (80.3)
Non-urgent	66 (34.6)	3.1 (2.1–6.6)	2 (3.0)	53 (80.3)
Non-indicated	64 (33.5)	4.7 (2.9–7.7)	0 (0.0)	52 (81.3)

*IQR* Interquartile range, *CTB* brain computed tomography

**Fig. 3:**
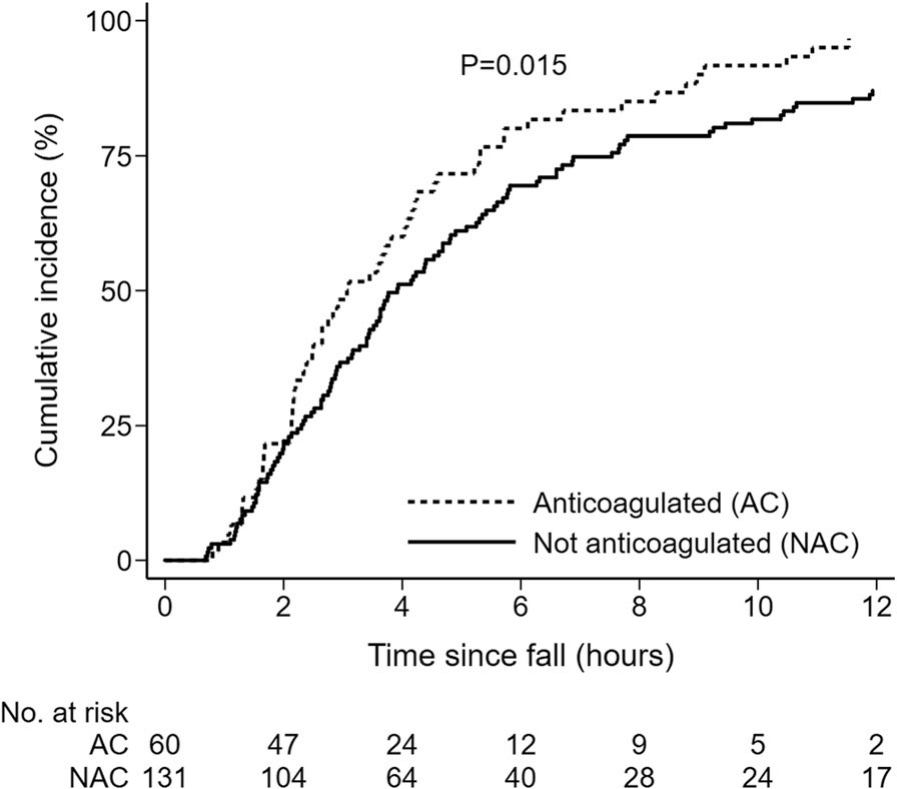
Cumulative incidence of brain CT by anticoagulation status in 191 patients after an inpatient fall

We found no evidence that imaging intervals were different between after-hours falls (8 pm-8 am) and in-hours falls (χ^2^ = 1.66, df = 1, *P* = 0.20). However, there was evidence that imaging intervals were shorter in patients on anticoagulants compared to those who were not (χ^2^ = 5.71, df = 1, *P* = 0.015), as shown in Fig. 3. The hazard ratio (HR) for performing a CTB in anticoagulated patients was 1.48 (95% CI: 1.09–2.02). This estimate was unchanged even allowing for the clinical urgency of CTB in multivariable Cox regression (HR = 1.49, 95% CI: 1.08–2.07, *P* = 0.016).

### Cerebral haemorrhage

The overall incidence of ICH was 0.9% (8/934). There were five subdural and three parenchymal haemorrhages. Our analysis showed that multi-level logistic regression models were not required. The factors associated with ICH by standard logistic regression are shown in Table 6. The factors not associated with ICH are summarised in Additional file 2: Table S1. Due to few ICH events, we could not build a multivariable model. There were also insufficient events to use the Mantel-Haenszel stratification method to adjust for confounding (Mantel-Fleiss criterion < 5). None of the patients with ICH had coagulopathy or intracranial malignancy, so no risk estimates were possible.

**Table 6:** Unadjusted logistic regression for intracranial haemorrhage after an inpatient fall (*N* = 934)

Variable	OR	95% CI	*P*
Anticoagulation	4.09	1.01–16.6	0.049
CKD eGFR < 30 ml/min/1.73m^2^	5.37	1.26–22.9	0.023
Head strike	23.1	2.83–188.6	0.003
Lost consciousness or amnesia	23.5	5.26–105.4	< 0.001
Drop in GCS > 2^a^	58.7	13.3–260.2	< 0.001

*OR* Odds ratio, *GCS* Glasgow Coma Scale, *CKD* Chronic kidney disease

^a^Data missing in 6.0% of falls

### Outcomes following imaging

In 183/191 (95.8%) falls without ICH on CTB, 175/183 (95.6%) did not experience a change in clinical management, 8/183 (4.4%) had a medication change or reversal of anticoagulation, and none received palliative therapy. In 8/191 (4.2%) of falls associated with ICH, 2/8 (25%) had no change to management, 4/8 (50%) had a medication change or reversal of anticoagulation, and 2/8 (25%) were palliated. There were no patients who received neurosurgical intervention following CTB. There was strong evidence that the result of the CTB changed clinical management (*P* < 0.001).

## Discussion

This study found that falls in hospital acute medical wards resulted in a much lower ICH incidence of 0.9% compared to community falls applying the CCTHR, which was estimated at 5 to 8% [6–10]. Falls occurred constantly at all hours on the medical wards but the majority were low risk for head injury. We found that prioritisation of CTB was associated with anticoagulation status, rather than the clinical assessment of urgency. Most urgent CTB could not be performed within one hour of the fall but was not influenced by the time of day the fall occurred. We identified the factors associated with ICH as head strike, drop in GCS, loss of consciousness or amnesia, therapeutic anticoagulation and an eGFR < 30 ml/min/1.73m^2^.

Our estimate of inpatient ICH incidence is similar to a tertiary Japanese hospital of 1.2% [12]. There are several reasons which may explain the lower incidence. Firstly, there were many low-risk falls. Only half of falls occurred from standing height and only one in four had head strike appreciated. In contrast, high impact or high energy injuries from road accidents, violence and sports are common in ED. [10] Amongst Australians aged 65–69, 51% of fall-related hospitalisations were due to falls from ladders, stairs and steps in 2014–15 [23]. Thus, the mechanism of injury and impact force could explain some of the difference. This is supported by an ED study which found a lower incidence (3.5%) of ICH when the analysis was limited to ground-level falls in patients on antithrombotic agents [13]. Secondly, the denominator (those at risk for the outcome) was not limited to patients with head strike, unlike many ED studies. About 70% of our falls were unwitnessed, and one in five patients had premorbid cognitive impairment or delirium. Information on head strike, loss of consciousness or worsening confusion as indicators of head injury can be difficult to ascertain in such patients. This contrasts with ED studies which mostly included patients with clear head injury as the denominator. Thirdly, most patients with minor injuries after a community fall do not present to ED but we expect that all inpatient falls regardless of severity are captured by the incident reporting system. These key differences between community fallers and inpatient fallers suggest we should consider them as two distinct study populations.

Another important finding of this study was that guideline-based urgent CTB was not performed in a timely manner, and did not appear appropriately prioritised. Anticoagulation history appeared to be a main driver for expedited imaging. Patients who fell while on anticoagulants were 1.5 times more likely to receive a CTB at any given interval post-fall, compared to patients not on anticoagulants. This association was independent of urgency status based on guidelines. We suspect this may have arisen from a lack of standardised documentation of urgency on the CTB request form, where anticoagulation status became a surrogate indicator of urgency. It is possible that knowledge of the anticoagulation status created a sense of fear or anxiety about ICH leading to a subjective urgency that overrode the risk assessment, with quicker ordering and completion of the CTB.

NICE guidelines suggest that urgent CTB should be performed within one hour [9]. This was not achieved for most patients for many possible reasons: delayed post-fall clinical assessment, delayed development of neurological symptoms/signs or changes in GCS, failure to recognise or document urgency in the imaging request, service delivery, and logistic issues. It is also unclear if the one hour timeframe should begin from the fall event, time of post-fall assessment, clinical deterioration, or when the decision to scan is made. The hospital guidelines do not specify this and there is no standard metric to monitor this. We believe that development of hospital-specific guidelines in this area would be useful. In this study, we have used the time of fall as the beginning of the imaging interval.

This study supports the value of formal GCS monitoring post fall, in line with the AHEAD study of 3566 older adults on anticoagulants who presented to ED following blunt head injury [14]. We found that a drop in GCS and post-fall loss of consciousness/amnesia were associated with detection of ICH on CTB. These changes in mental status may represent related phenomena and we could not determine if both were independently associated with the outcome. However, another study in patients with head injury on antithrombotic medications showed that loss of consciousness at the time of fall predicted ICH on CTB, independent of a normal CGS at the time of presentation to ED. [16]

Head strike and anticoagulation were also associated with a higher risk of ICH, while antiplatelet medications, either single or combined, were not. This is in contrast to a meta-analysis in 2017 which showed that with the exception of aspirin monotherapy, antiplatelet use was associated with traumatic ICH, with an odds ratio of 1.87 (95% CI: 1.27–2.74) [24]. However, the heterogeneity of the included studies makes it difficult to directly compare those results with our study. The association of age and dementia with post-fall ICH is also unclear [10, 14, 16]. Older age and dementia may be risk factors for traumatic ICH as cerebral atrophy is correlated with ICH after head trauma in patients over 60 years old [25]. We found no association between age and ICH, which is consistent with the findings of the AHEAD study [14].

This study also found an association between ICH and advanced CKD. On average, patients with an eGFR< 30 ml/min/1.73m^2^ had a five times higher risk of ICH following an inpatient fall than those with an eGFR≥30 ml/min/1.73m^2^. The association between advanced CKD and traumatic ICH could be potentially explained by platelet dysfunction [17, 18]. However, therapeutic anticoagulation may be a confounder of this association as anticoagulation was correlated with both ICH and advanced CKD. It was reported that long term haemodialysis patients receiving frequent anticoagulation for dialysis may develop spontaneous subdural haematomas [26]. The small number of patients with advanced CKD and ICH in this study limits our ability to draw firm conclusions.

### Limitations

The limitations of our study included our inability to conduct a multivariable analysis to control for confounders due to the low incidence of ICH. Given the retrospective nature of the study, 12.1% of eligible falls were excluded due to poor documentation in the medical record. It was unclear if this has biased the analysis. We do not believe that falls were under-reported in the RISKMAN database as we had to exclude 5.9% of eligible falls as “near-misses”, suggesting that over-reporting was more likely.

Given the existing local imaging criteria, there may be a selection bias. We may have missed asymptomatic ICH by not performing universal post-fall imaging, and underestimated the incidence. However, universal imaging is not performed in head trauma patients presenting to ED either. Furthermore, defining the denominator for the incidence remains difficult. We did not exclude patients without a definitive head strike which may also have contributed to a lower incidence.

It is also possible that some falls were secondary to a spontaneous ICH, rather than being primarily traumatic. However, spontaneous subdural bleeds are rare, comprising only 2.6% of all subdural bleeds in a neurosurgical study [27]. Chronic dialysis patients may be an exception due to frequent anticoagulant exposure [26]. We also established that none of the cases of ICH had an admission diagnosis of stroke to suggest subsequent haemorrhagic transformation. However, there were two patients with parenchymal ICH where we could not exclude the possibility that spontaneous ICH (e.g. due to amyloid angiopathy) was the primary event leading to the fall.

There was missing data for GCS in 6.0% of falls due to non-adherence to protocol. It is possible that more CTB may be performed if these observations were not missing. This is difficult to delineate as there is more than one indication to request a CTB. As we did not use a multivariable model, the missing GCS would not affect other univariable analyses, so these cases were not excluded. BMI could not be calculated in 22% of patients. A logistic regression analysis for ICH on BMI could not performed as it would be unreliable. However, a previous study indicated that obesity was not a determinant of severity of traumatic brain injury in low level falls [28]. Four percent of falls did not progress to CTB despite an indication. These were situations where clinical judgement was at odds with guidelines but the reasons were not explored in this study. Frailty, life expectancy and consent may be relevant factors.

### Implications for practice and research

The CCTHR and NICE guidelines rely significantly on clinical evidence or history of blunt head trauma and neurological symptoms, to define risk for brain injury on CTB. In acute medical wards, patients with cognitive impairment or delirium who have an unwitnessed fall sit in a grey area for risk assessment due to the difficulty in confirming actual versus suspected blunt head trauma, and differentiating genuine neurological alterations from abnormal baseline function. Our adapted guidelines do not require specific determination of head strike in unwitnessed falls for recommending CTB, if there are neurological consequences. This approach may be contributing to excess CTB and refining the guidelines for these patients is essential.

We believe that community based head injury guidelines are based on a different study population than patients who fall on our medical wards. The WHO criteria provides a broad definition of a fall, resulting in the reporting of many low risk events where “dangerous mechanisms” are not involved. Our findings support the need for research examining the risk of ICH specifically arising from falls, incorporating information on the falls event such as the fall height. Any additional risk for ICH from antiplatelet therapy post-fall needs to be further elucidated but therapeutic anticoagulation seems to be a consistent risk factor. There was also a signal to consider novel risk factors such as advanced CKD. Finally, we question how the timing of CTB recommended by NICE guidelines should be applied and monitored in hospitalised patients. This is pertinent for health services as we found that most CTB deemed “urgent” could not be done within one hour. It is also unclear if failure to adhere to these imaging guidelines is associated with a worse outcome.

Since ICH from inpatient falls are rare, large studies of several thousand falls and several hundred CTB will be required to capture adequate events to allow a robust multivariable analysis of potential risk factors for ICH. For this purpose, collaboration between health services organizations and networks will be required. We encourage international bodies such as the International Hospital Federation or neurological associations to facilitate reporting of these incidents and outcomes in hospitals globally, so that a meta-analysis can be performed. To facilitate meta-analysis, the hospital community will need clear criteria for inpatient falls assessment and outcomes, and consistent reporting.

## Conclusions

We discovered issues in adopting an ED-based head injury guideline for inpatient falls. Most inpatients falls were low risk for head injury, and the incidence of ICH was lower than falls and head injury presenting to ED. Further research is required to make specific imaging guidelines for inpatient falls to minimise the risk of missing ICH, while avoiding unnecessary scans and optimising timeliness.

## Supplementary information

**Additional file 1: Figure S1.** Monash Health post-fall management form detailing the mandatory medical assessment and indications for brain CT.

**Additional file 2: Table S1.** Univariable logistic regression of other factors potentially associated with intracranial haemorrhage.

## Data Availability

The datasets used and/or analysed during the current study are available from the corresponding author on reasonable request, subject to approval by the Monash Health Research Directorate and Quality Unit.

## Abbreviations

BMI: Body mass index
CCTHR: Canadian CT head rules
CKD: Chronic kidney disease
CTB: Computed tomography of the brain
ED: Emergency Department
eGFR: estimated glomerular filtration rate (based on CKD-EPI equation)
GCS: Glasgow Coma Scale
HR: Hazard ratio
ICH: Intracranial haemorrhage
INR: International normalised ratio
NEXUS II: National Emergency X-Radiography Utilisation Study II
NICE: National Institute for Health and Care and Excellence
WHO: World Health Organisation
