# Supplementary material for: Intracranial haemorrhage detected by cerebral computed tomography after falls in hospital acute medical wards
Source: BMC Health Serv Res. 2019 Nov 4;19:792. doi: 10.1186/s12913-019-4634-8 (PMC6829924; doi:10.1186/s12913-019-4634-8)
Supplement: Supplementary file 1 — Additional file 1: Figure S1. Monash Health post-fall management form detailing the mandatory medical assessment and indications for brain CT. [file 12913_2019_4634_MOESM1_ESM.pdf]

IDENTITY

MonashHealth

- ☐ Dandenong Hospital
- ☐ Monash Medical Centre Clayton
- ☐ Kingston Centre
- ☐ Moorabbin Hospital
- ☐ Jessie McPherson
- ☐ Community Health Services
- ☐ Casey Hospital
- ☐ Cranbourne Integrated Care Centre

Affix Patient Identification Label

Unit Record Number: ....

Surname: ....

Given Name: ....

D.O.B: .... Age: .... Sex: ....

Address: ....

....

SHF04270V

POST FALL MANAGEMENT FORM

Post Fall Management Form to be used for **ALL Falls.**
This post fall management plan should not replace clinical judgement.
To be used in conjunction with the post fall management procedure.

- * Page 1 to be completed by nursing
- * Page 2, 3 & 4 (part A) to be completed by medical
- * Page 4 (part B) to be completed by interdisciplinary

PART A: Undertake investigations as clinically indicated by their injuries or fall mechanisms.

Site:	Ward:	Date of fall:	Time of fall:
Time patient found:	Time medical notified:	Date of medical review:	Time of medical review:

SITUATION

Please circle

DETAILS OF THE FALL

Witnessed / Unwitnessed

If witnessed, witnessed by

Head strike: YES / NO / SUSPECTED / UNSURE

LOC: YES / NO How long? ______

Where did the fall occur? ______

Type of fall: Slip / Trip / Bumped into object / fall onto object / Transfer/other (specify) ______

Activity being undertaken ______

______

Position of patient ______

Patient recall of fall ______

______

______

PATIENT

Alert/Orientated/Confused/Agitated/Impulsive/Other (specify) ______

Does the patient lack insight in their risk of falling: YES / NO

Last toileted at: ______

Was the patient incontinent at the time of the fall? YES / NO

Was the patient wanting to go to the toilet at the time of the fall? YES / NO

Restraints in use at time of fall? YES / NO

Specify restraints used ______

Any changes in medication in the past 24 hours? YES / NO. If yes, what changes ______

______

INTERVENTIONS IN PLACE IMMEDIATELY PRIOR TO FALL

Patient able to follow instructions	YES / NO
Instructions on calling nurse prior to fall?	YES / NO
Call bell within reach	YES / NO / NA
Brakes on bed	YES / NO / NA
Bedrails	UP / DOWN / NA
LoLo bed position at time of fall	UP / DOWN / PART / NA
Bed/chair at suitable height	YES / NO / NA
Chair stable	YES / NO / NA
Bedside table within reach	YES / NO / NA
Gait aid within reach	YES / NO / NA
Eyewear in use or within reach	YES / NO / NA
Personal items within reach	YES / NO / NA
Appropriate footwear	YES / NO
Appropriate clothing	YES / NO
Assisted with ambulation/transfers	YES / NO / NA
Adequate nutrition & hydration	YES / NO
Family member present	YES / NO

ENVIRONMENT

Room sufficiently well lit	YES / NO
Floor hazards (e.g. wet, slippery)	YES / NO
Room free from clutter	YES / NO

Visibility of patient at time of fall
VISIBLE / NOT VISIBLE / DOOR CLOSED / CURTAINS DRAWN / OTHER (specify) ______

FALLS RISK ASSESSMENT (prior to fall)

LOW MEDIUM HIGH

Date last completed:

☐ Riskman entered. Riskman ID No.______

Are there any injuries sustained? YES / NO / SUSPECTED
Specify injuries sustained or suspected______
Were all documented prevention strategies (as per MRI33) in place at the time of the fall? YES / NO
If identified strategies were not in place, list these below.

Other information (include contributing factors to the fall):			
Print name:	Signature:	Designation:	Date & time:

POST FALL MANAGEMENT

MEDICAL REVIEW POST FALL IS MANDATORY
The timing of the review depends on the clinical situation:

- **If there is a decrease of ≥ 2 on the Glasgow Coma Scale the medical review must occur immediately and a MET called.**
- **If injuries are obvious or suspected, or the patient is at increased risk of bleeding, medical review must occur within an hour**
- **All other patients must be reviewed within 4 hours**

Medical Officer (print name & pager):	Date & Time of review:

SHF04270V

BACKGROUND

LOC: Y/N Unwitnessed: Y/N Delirium: Y/N Dementia: Y/N Toilet related: Y/N

Amnesia and duration:

Confirm circumstances of the fall (as documented on page 1 and with discussion with nurse) and document any variations or additional information.

ASSESSMENT

Increased bleeding risk:

Do any of the following apply to the patient?

☐ Bleeding disorder

☐ Anticoagulant therapy, include medications such as warfarin, dabigatran, apixaban, rivaroxaban, and therapeutic doses of heparin and enoxaparin

☐ Antiplatelet medications such as clopidogrel, prasugrel or ticagrelor

Medications Risks (circle): Diuretics / antihypertensives / sedatives / narcotics / antipsychotics

FWT (circle): Nitrates / leucocytes / not done

BP lying (manual)	Temperature	GCS
BP standing (manual)	Oxygen saturation	AMTS
HR	BGL	
RR	JVP	

Head: Hips:

Neck: Legs:

Arms: Other Injury:

Tone **Muscle strength- please record +/-**

	R	L		R	L
Normal			Hip Flex	/5	/5
Increased			Hip Ext	/5	/5
Parkinsonian			Hip Abd	/5	/5
			Ankle PF	/5	/5
			Ankle DF	/5	/5

Medical Examination

Examine patient with particular focus on:

- **Postural hypotension, neurological status, musculoskeletal and cutaneous injuries**
- **Symptoms of intracranial pressure such as severe headache and vomiting,**
- **Signs of base of skull fracture such as periorbital and mastoid ecchymosis, CSF rhinorrhoea, bleeding from the nose or ears and haemotympanum.**

Affix Patient Identification Label

Unit Record Number: ....

Surname: ....

Given Name: ....

D.O.B: .... Age: .... Sex: ....

Address: ....

....

ASSESSMENT continued

Reflexes

	R	L
Biceps		
Br rad		
Knee		
Ankle		
Plantar		

Cranial nerves—screen

	R	L
Pupils		
Ocular ROM		
Facial droop		

Other examinations:

Does the patient look unsafe getting into/out of bed or chair or when walking? YES / NO

Injuries identified:

Risk factors identified: (please tick all relevant)

☐ Abnormal mobility (including unsteady gait)
☐ Increased risk of bleeding
☐ Postural hypotension
☐ Abnormal neurology
☐ Abnormal bone health → Vitamin D level: ____________
☐ Cognitive impairment
☐ Medications
☐ Previous falls
☐ Toileting/continence

REQUEST

PLAN

Indications for urgent CT:

☐ Unwitnessed fall and / or head strike with
  ☐ Altered GCS
  ☐ Suspected skull fracture
  ☐ Signs of raised ICP, or focal neurological deficit
  ☐ Signs of worsening confusion
  ☐ Any loss of consciousness or amnesia post fall
and are over 65 at increased risk of bleeding

Indication for CT within 8 hours

☐ Increased risk of bleeding with known or probable head strike

Indication for coagulation check:

☐ Patients on anticoagulants with a definite or probable head strike. Result must be discussed with Medical Registrar/Consultant regarding the need for reversal of anticoagulation.

Indications for cervical spine CT (must also have a collar applied)

☐ unwitnessed fall and / or head strike with any of posterior midline cervical tenderness, a focal neurological deficit, presence of a distracting injury.

Neurological observations:

☐ Unwitnessed fall or head strike
- 15 minutes for 1 hour, then
- Every 30 minutes for 4 hours, then review

☐ Patient is currently on Anticoagulant therapy, include medications such as warfarin, dabigatran, apixaban, rivaroxaban, and therapeutic doses of heparin and enoxaparin
- 2 Hourly for 24 hours, then
- 6 Hourly for 24 hours

☐ XRay (indicate body part/s for xray)
☐ Postural blood pressure
  Frequency of postural blood pressure: ___________
☐ INR
☐ APPT
☐ ECG
☐ MSU
☐ Septic screen
☐ Falls risk assessment (MR133) updated
☐ Medication review completed
☐ Family / NOK informed of fall and post fall management plan
☐ Other, specify.

Request continued – see back page

MonashHealth

- ☐ Dandenong Hospital
- ☐ Kingston Centre
- ☐ Jessie McPherson
- ☐ Casey Hospital
- ☐ Monash Medical Centre Clayton
- ☐ Moorabbin Hospital
- ☐ Community Health Services
- ☐ Cranbourne Integrated Care Centre

Affix Patient Identification Label

Unit Record Number: ....

Surname: ....

Given Name: ....

D.O.B: .... Age: .... Sex: ....

Address: ....

....

REQUEST

Medications to be changed: ....

Analgesia required: ....

Interventions requested: (please tick requests)

- ☐ line of sight
- ☐ LoLoBed
- ☐ Regular toileting – separate from rounding
- ☐ CPO
- ☐ Bed rails up / down
- ☐ Hard Collar
- ☐ Other ....

Follow up by Registrar: Name: .... Date: ....
Time: ....

PART B: POST FALL FOLLOW UP—An interdisciplinary post fall review is to be conducted within 24 hours.
Date and time of review by team:

Review teams description of the fall (include contributing factors).

Interventions put in place immediately post fall:

- ☐ Line of sight
- ☐ LoLoBed
- ☐ Regular toileting– separate from rounding
- ☐ CPO
- ☐ Bed rails up/down
- ☐ Hard collar
- ☐ Other..........

☐ Mobility assessment conducted and documented in progress notes

☐ Falls assessment (MRI33) reviewed and updated as required

☐ Investigations conducted and results actioned

Additional interventions put in place post interdisciplinary review:
....

Has an injury been confirmed? YES / NO. Specify injury

Review team in attendance (print name, position, signature)

MEDICAL:

ALLIED HEALTH:

NURSING:

OTHER:
