# Supplementary material for: Intracranial haemorrhage detected by cerebral computed tomography after falls in hospital acute medical wards
Source: BMC Health Serv Res. 2019 Nov 4;19:792. doi: 10.1186/s12913-019-4634-8 (PMC6829924; doi:10.1186/s12913-019-4634-8)
Supplement: Supplementary file 2 — Additional file 2: Table S1. Univariable logistic regression of other factors potentially associated with intracranial haemorrhage. [file 12913_2019_4634_MOESM2_ESM.docx]

**Table S1. Univariable logistic regression of other factors potentially associated with intracranial haemorrhage (N=934)**

| **Variable** | **OR** | **95% CI** | **P** |
| --- | --- | --- | --- |
| Age (years) | 1.04 | 0.95 – 1.13 | 0.43 |
| Female sex | 1.21 | 0.29 – 5.12 | 0.79 |
| Hypertension | 2.79 | 0.56 – 13.9 | 0.21 |
| Chronic liver disease | 2.31 | 0.28 – 19.3 | 0.44 |
| Platelets <50,000/µL | 8.67 | 0.99 – 75.7 | 0.051 |
| Antiplatelet medication  None  Single  Dual | 1.00  0.34  1.71 | Reference  0.04 – 2.88  0.20 – 14.6 | 0.50 |
| Dementia | 1.43 | 0.28 – 7.20 | 0.66 |
| Unwitnessed fall | 2.92 | 0.34 – 23.7 | 0.32 |
| Fall from standing height | 0.50 | 0.12 – 2.09 | 0.34 |

Notes: The odds ratio for intracranial cancer and coagulopathy were not estimable as there were no events.
